# Experimental Research on Electromechanical Properties of Multiple Contact Surfaces Copper Bulks under Normal Cyclic Loading and Variable Temperature

**DOI:** 10.3390/ma12233883

**Published:** 2019-11-24

**Authors:** Limei Liu, Jiangtao Yan, Keyang Wang, Yang Liu, Wurui Ta, Yuanwen Gao

**Affiliations:** 1 Department of Mechanics and Engineering Science, College of Civil Engineering and Mechanics, Lanzhou University, Lanzhou 730000, Gansu, China; liulm@lzit.edu.cn (L.L.); yanjt18@lzu.edu.cn (J.Y.); wangky17@lzu.edu.cn (K.W.); yliu18@lzu.edu.cn (Y.L.); tawr@lzu.edu.cn (W.T.); 2 Key Laboratory of Mechanics on Environment and Disaster in Western China, The Ministry of Education of China, Lanzhou University, Lanzhou 730000, Gansu, China

**Keywords:** contact resistance, cyclic load, temperature, number of contact surfaces

## Abstract

Contact resistance is key for stable operation of electrical contact equipment, and can also be extensively applied. For Tokomak devices in fusion reactors, contact resistance of the superconductor magnet system strongly relates to the alternating current (AC) loss of the cable; the cable is assembled using a certain number of contacting superconducting tapes coated with copper layers on both sides. The contact resistance of a metal solid surface is affected by many factors. In this work, the contact resistance of copper surface samples was studied experimentally under variable normal cyclic load, temperature and number of contact surfaces. This is consistent with real-world working conditions, as the structure of superconducting cables can be changed, and such cables are used under cyclic electromagnetic forces in temperatures which range from room to working temperature. Experimental results showed that contact resistance decreased rapidly with an increase of load. Further, when temperature was varied from 77 to 373 K, the load–unload contact resistance lag decreased. When the number of contact surfaces was increased, contact resistance increased. Finally, a fitted formula describing the relationship between contact resistance and cyclic times, temperature and number of contact interfaces was determined. This formula can be used to predict variation trends of contact resistance in complex environments and provide more accurate contact resistance parameters for calculating the AC loss of superconducting cables.

## 1. Introduction

Electrical contact resistance is key to stable operation of electrical equipment. It is also extensively applied in various fields, including in the creation of resistance spots on metal elements [1,2,3], making force sensors in micro-electromechanical systems [4], making gas sensors using contact between semiconductors [5], detecting critical points in the contact area of connectors [6], and obtaining a lubrication contact diagnosis between contact surfaces [7]. 

Used especially in the superconductor magnet system of Tokomak devices in fusion reactors, a new generation of non-insulated REBCO (Rare Earth-Barium-Copper Oxide) tape, which possesses a layer of copper coated on both sides, has been shown to have strong mechanical properties and high critical current density. In addition, REBCO magnets wound with these tapes have a self-quenching protection function [8,9,10,11]. When a non-insulated REBCO magnet actually works, internal current flows between the tapes. Transverse contact resistance between the tapes is a key parameter of the internal alternating current (AC) loss of the magnet. AC loss inside the magnet can only be calculated effectively when the contact resistance between the tapes under the action of cyclic load is accurately known. However, in actual working conditions, the electromagnetic force circulates to produce extrusion inside the magnet coil, which changes the contact resistance and causes current redistribution. Hence, the contact resistance of a cable strongly relates to its AC loss, as it is assembled using a certain number of contacting superconducting tapes. 

Since Holm [12] established the theoretical framework of electrical contact, many scholars have used fractal geometry [13], finite element analysis [14,15], empirical modeling [16], and other methods to study factors which influence the electrical contact between metal solids. Such factors include the metal surface morphology [17,18], surface films [13,15,19], mechanical properties (hardness and elastic modulus) [3,17], temperature [20,21,22], and cyclic loading [13]. The results of these studies have shown that contact resistance is composed of constriction resistance and surface film resistance [12]. In addition, constriction resistance has been found to be related to surface topography, and decreases with an increase in fractal dimension and load [13]. It has been reported that, when an oxide film exists, the smaller the surface roughness value, the larger the contact resistance [13,15]. During cyclic loading, contact resistance increases with an increase of the loading period [10]. Further, when the hardness of a material increases, its plastic deformation lags [3]. At liquid nitrogen temperature, the contact resistance of metal conductors is several to more than 1000 times that at normal temperature [20,21].

Although a great deal of research has been performed on the factors influencing contact resistance, there is little research on the relationship between contact resistance and force under normal cyclic load with multiple surfaces and variable temperature. Therefore, it is significant to study multi-interface contact resistance of copper bulks under cyclic loading and variable temperature.

In this work, the effects of the number of contact surfaces, temperature, cyclic load and number of cycles on contact resistance were studied experimentally.

## 2. Experiment

**Figure 1:**
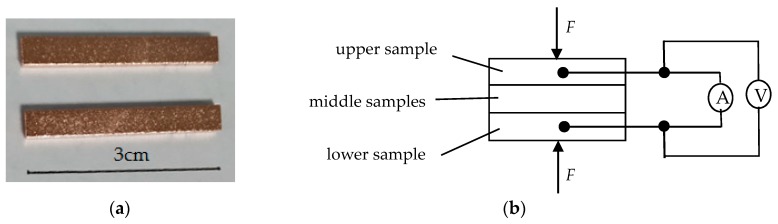
Samples and principle of resistance measurement by the four-wire method. (**a**) Samples and (**b**) measurement principle.

The sample material was T2 copper with 99.90% copper content. The sheet was cut into blocks 30 mm × 4 mm × 2 mm in size by wire-cut electrical discharge machining. Uniform surface micro-morphology was obtained by shot blasting. Surface oxide layers and oil stains were removed by a copper bright cleaning agent, after which the samples were cleaned by pure water and dried in air. Contact resistance was measured according to the four-wire method. The samples and measurement principle are shown in Figure 1.

**Figure 2:**
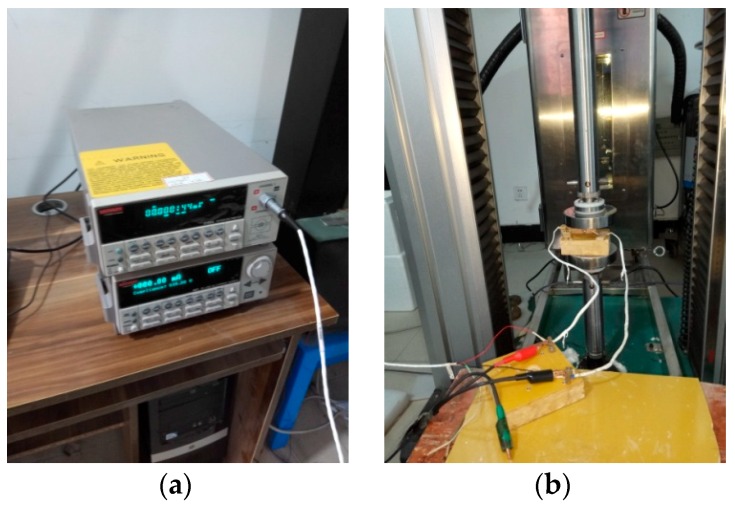
Measuring devices. (**a**) KETHLEY-2182A nanovoltmeter and KEITHLEY-6221 current source; and (**b**) UTM4304HB electronic universal tester and high–low temperature chamber WGDN-19150S.

The measuring devices are shown in Figure 2. A KETHLEY-2182A nanovoltmeter, KEITHLEY-6221 current source, UTM4304HB electronic universal tester (1 kN sensor, error in range ± 0.5%) and high–low temperature chamber WGDN-19150S (error ± 0.5 °C) were adopted.

During the experiment, the nanovoltmeter was set to zero, and then the installed sample was loaded in the normal direction with the electronic universal tester. After the specified load was reached, the current supplied by the current source was increased from 0.005 A to 0.1 A. Voltage and current at 30 measuring points were measured, and then the resistance was calculated based on the voltage–current curve. The load was loaded for 10 cycles in the range of 6 to 960 N, and the voltage value, current value and contact resistance were measured and calculated under different cycles of loads, specifically 6 N, 12 N, 24 N, 48 N, 96 N, 156 N, 240 N, 480 N and 960 N. During the experiments with variable temperature, five pieces of superimposed sample were installed in the high–low temperature chamber, and its temperature was set to a specific value for 30 min. This was to ensure that the temperature of the samples, conductors and other parts were fully consistent with the temperature of the chamber. Then, voltage and current under the set load value of different cycles were measured repeatedly, and the measured resistance obtained. In addition, the number of samples was changed. Specifically, the number of intermediate samples was changed from 0 to 1, 3, 6 and 10, leading to 1, 2, 4, 7 and 11 contact surfaces, accordingly. The voltage and current of the set load value in different cycles were again measured and used to obtain the measured resistance.

## 3. Results

In the measurement of contact resistance, the total resistance, R, contained the conductor wire resistance, $R_{01}$, bulk resistance of the samples, $R_{02}$, resistance of the joints, $R_{03}$, and resistance of the contact surfaces, $R_{c}$. In other words, $R=R_{01}+R_{02}+R_{03}+R_{c}$, where $R_{01}$, $R_{02}$ and $R_{03}$ were constant values because the conductor wire, size of the Cu samples and mode of joints were invariant. It was assumed that contact resistance was $R_{0c\min}$ under the maximal normal force during the first loading, so that $R_{0}=R_{01}+R_{02}+R_{03}+R_{0c\min}$, where $R_{0}$ was the total resistance under the maximal normal force during the first loading. Then, under other normal forces:

(1) $$R=R_{0}+R_{c}-R_{0c\min}$$

If relative contact resistance, $R^{\;*}$, is defined as the measured resistance, R, divided by $R_{0}$, then,

(2) $$R{\;}^{*}=R/R_{0}=1+\left(R_{c}-R_{0c\min}\right)/R_{0}$$

We experimentally studied the relationship between relative contact resistance $R{\;}^{*}$ and normal load F as the number of contact surfaces, temperature, load and number of cycles was varied.

### 3.1. Results of Contact Resistance of a Single Contact Surface at Room Temperature

**Figure 3:**
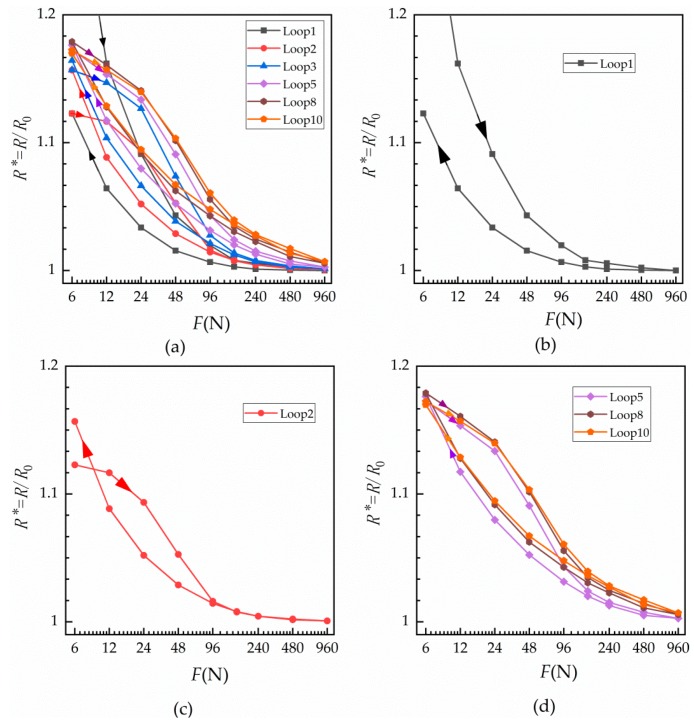
Normal cyclic load against relative contact resistance of a single contact surface at room temperature. Relative contact resistance curves obtained under the given normal loads during (**a**) the first, second, third, fifth, eighth and tenth cyclic loadings; (**b**) the first cycle only; (**c**) the second cycle only; and (**d**) the fifth, eighth and tenth cycles.

The relationship between relative contact resistance and normal load for a single contact surface under cyclic loading at room temperature was measured and is shown in Figure 3.

From Figure 3, it can be seen that relative contact resistance seems to decrease exponentially with an increase of load. Further, the relative contact resistances do not appear to be equal during loading and unloading. In the first loading cycle, shown in Figure 3b, contact resistance was greater under loading than the unloading contact resistance, and the resistance changed quickly under a small load. However, when the load exceeded 96 N, contact resistance changed only slightly. Figure 3c shows the curve for the second cyclic loading. It can be seen from the figure that the resistance after unloading is greater than that at loading when the load returns to 6 N, while the resistances of loading and unloading after 96 N are basically the same. In Figure 3d, with the increase in the number of cycles, the resistance does not change with the number of cycles after the eighth loading.

**Figure 4:**
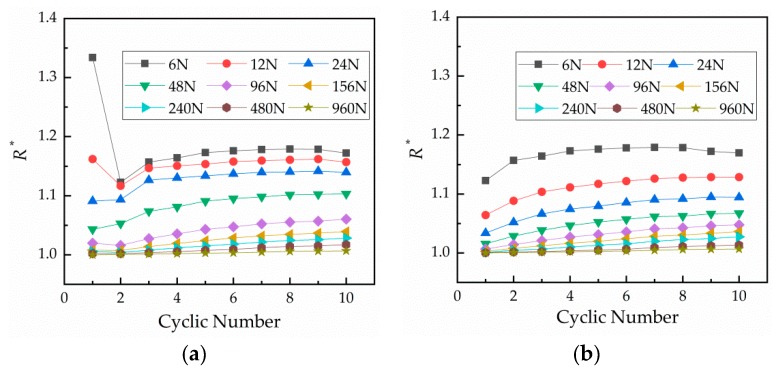
Relationship curves between the number of cycles and relative contact resistance under different loads. (**a**) Curves under loading and (**b**) curves under unloading.

The relationship between resistance and number of cycles under different loads is shown in Figure 4, which reflects the change of contact resistance with the number of cycles. Figure 4a displays the loading curves and Figure 4b shows the unloading curves. Contact resistance increases with the number of cycles under each load and then tends to stabilize. The first loading is unstable under small loads.

At loading and unloading, the force–relative contact resistance curves did not coincide. Under the action of the same normal force, the fact that the unloading contact resistance was less than the loading contact resistance indicates that the actual conductive area during unloading was greater than during loading. The reason for this is that the actual conductive area increases with the contact area—there are more micro-plastic and elastic deformations as the normal load increases, increasing the contact area. At the unloading stage, the actual conductive area of unloading was larger than that of loading because the adhesion applied on the contact and material surfaces easily oxidized in the air. With the increase of cycles, the actual conductive area decreased as the surfaces progressively oxidized and hardened, thereby increasing contact resistance $R_{c}$.

### 3.2. Results of Force–Relative Contact Resistance under Variable Temperature

In real-world applications, electrical contact equipments are sometimes used at different temperatures. For example, the magnet system manufactured in the new generation of non-insulated high-temperature superconducting strips involves contact between two Cu surfaces or a Cu surface and another metal surface [11]. So, we completed experiments at different temperatures.

**Figure 5:**
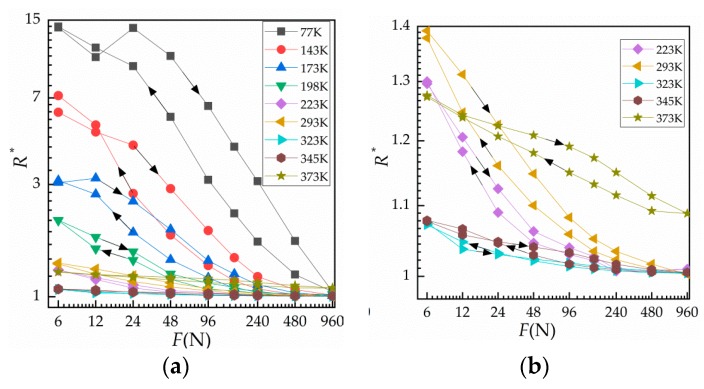
Normal load against relative contact resistance in a variable temperature environment. (**a**) Curve of $R{\;}^{*}$ against load at different ambient temperatures from 77 to 373 K and (**b**) curve of $R{\;}^{*}$ against load at different ambient temperatures from 223 to 373 K.

The results obtained by measuring the contact resistance of five contact samples at temperatures ranging from 77 K (liquid nitrogen temperature) to 373 K (heated to 100 ℃) under 10 cycles of loading are shown in Figure 5. Figure 5a shows the load–relative contact resistance curves for all given temperatures from 6 to 960 N during the third loading–unloading cycle. Figure 5b is an enlargement of the load–relative contact resistance curves for 223–373 K. In the figure, at a given temperature, the resistance at loading is greater than that at unloading, which is due to the adhesion between surfaces during unloading. As the temperature rises, the difference becomes insignificant. However, at 373 K, the change of load–unload resistance is higher than that at 345 K, which is due to the increase of temperature and the acceleration of surface oxidation.

**Figure 6:**
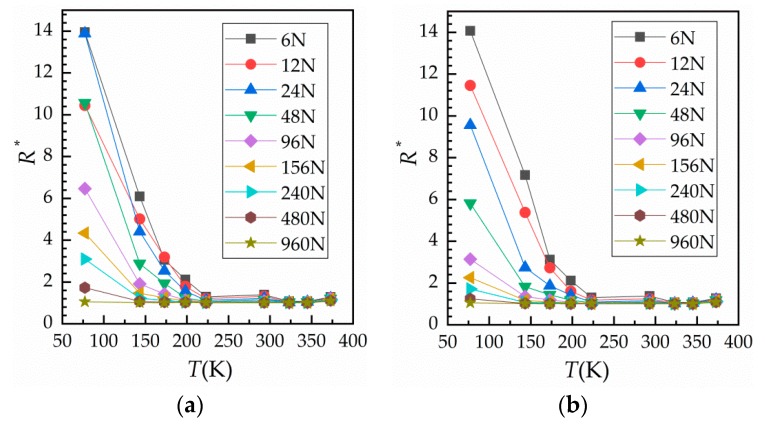
Curves of temperature against relative contact resistance. (**a**) During loading and (**b**) during unloading.

Using the results obtained for Figure 5a, the relationship between temperature and contact resistance was gained and is shown in Figure 6. Figure 6a shows the change of contact resistance with temperature during loading, while Figure 6b shows the change of contact resistance with temperature during unloading. It can be seen that contact resistance initially decreases rapidly with an increase in temperature; however, when the temperature exceeds 223 K, contact resistance does not change much. Although the resistivity of Cu and bulk resistance both decrease at low temperatures, contact resistance actually increases as contact area and conductive area become smaller due to hardening of the surface. This seriously affects the reliability of electrical devices working at low temperatures.

### 3.3. Results of Force–Relative Contact Resistance as the Number of Contact Surfaces Changes

**Figure 7:**
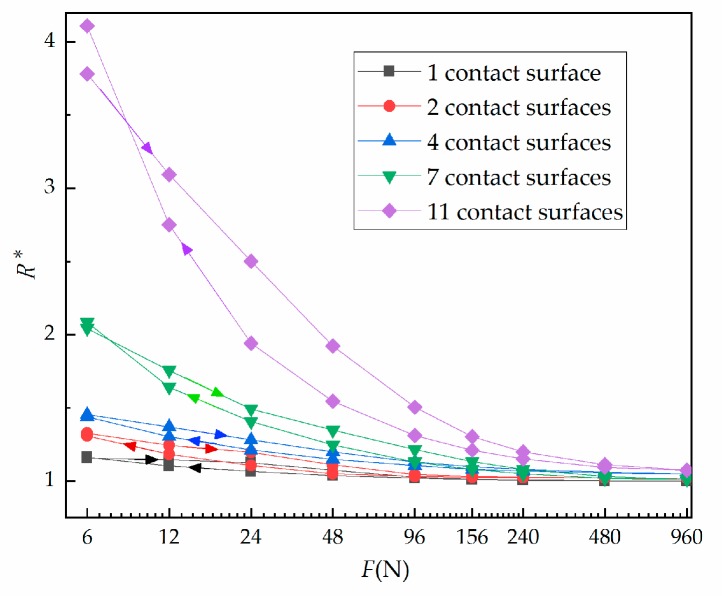
Third loading and unloading cycle showing the force against relative contact resistance under varied contact surface number.

**Figure 8:**
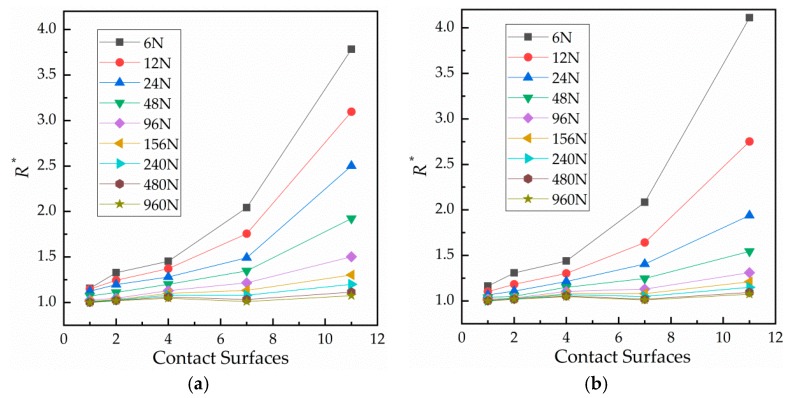
Curves of the number of contact surfaces against relative contact resistance. (**a**) During loading and (**b**) during unloading.

The influence of the number of contact surfaces on contact resistance is shown in Figure 7. Specifically, Figure 7 shows the relationship between relative contact resistance and normal load during the third cycle with 1, 2, 4, 7 or 11 contact surface(s). It is clear that when the number of contact surfaces is fixed, contact resistance decreases with an increase of load. In addition, the contact resistance at loading is greater than that at unloading. The more contact surfaces, the greater the change. Figure 8 shows the relationship between contact resistance and number of contact surfaces. When the load is fixed, contact resistance increases as the number of contact surfaces increases. Figure 8a shows the loading condition, while Figure 8b shows the unloading condition.

## 4. Discussion

In order to reveal the effects of the cycle number, environment temperature, and number of interfaces on contact resistance under normal cyclic loading–unloading, we used the following formula to fit the experimental results curves:

(3) $$R^{\;*}=y_{0}+Ae^{BF}$$

where $R^{\;*}$ is the relative contact resistance, $R{\;}^{*}=R/R_{0}$, $y_{0}$, A and B are the fitting parameters, and F is normal load in units N.

### 4.1. Fitting and Discussion of the Influence of Cyclic Number on Contact Resistance

**Figure 9:**
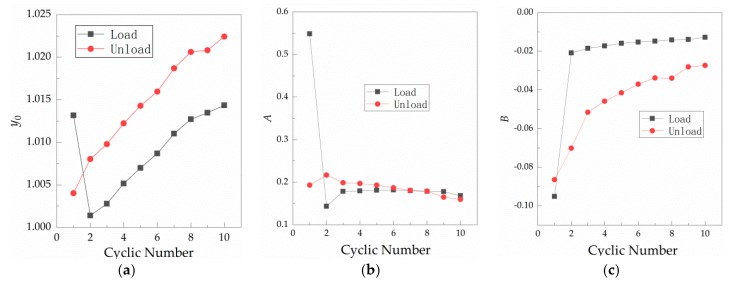
Variation in the fitting parameters with the number of cycles of a single contact surface at room temperature. (**a**) Curve of $y_{0}$ against the cycle number of loading and unloading; (**b**) curve of A against the cycle number of loading and unloading; and (**c**) curve of B against the cycle number of loading and unloading.

The experimental data in Figure 3 were fitted by Equation (3), and the fitting results are shown in the Table 1:

From Table 1, it can be seen that the range stays above 0.94 under all the cycles, indicating that Equation (3) fits well with the normal cyclic load against relative contact resistance relationship shown in Figure 3a. Figure 9 shows the relationship between the fitting parameters $y_{0}$, A and B of Table 1 and number of loading cycles.

From Table 1 and Figure 9, it can be found that $y_{0}$, A and B become unstable when the force is loaded in the first load, which is consistent with actual initial contact instability. From the first unloading, it is in a stable state of change. Figure 9a shows that, in the stable state, $y_{0}$ under loading is larger than that under unloading. Under loading and unloading, $y_{0}$ increases linearly with the number of cycles; i.e., the maximum variation of $y_{0}$ during loading and unloading is 0.013 and 0.0144, respectively, which only accounts for 1.28% and 1.41% of the maximum value of $y_{0}$. This essentially indicates that $y_{0}$ is a fixed value under a single contact surface, which may be caused by oxidation of the copper surface after exposure to air. Changes in A are shown in Figure 9b. In the stable state, A decreases gradually with the number of cycles during unloading, and its maximum change is 0.0572 or 26.4% of the maximum value of A during unloading. During loading, it remains stable with the number of cycles, with the maximum change of 0.0384 or 21.1% of the maximum value of A under loading. The maximum change of loading and unloading A is 0.0347 or 16.01% of the maximum value of A during unloading. Changes in B are shown in Figure 9c. In the stable state of contact, the loading fitting index B is larger than that under the unloading condition. Both loading and unloading fitting index B increase with the number of cycles, and then tend to stabilize. B changes from −0.0209 to −0.0128 with an increase of 0.0081, or 38.76% of 0.0209. Under unloading, B increases from −0.0702 to −0.0274, or 60.97% of 0.0702. According to Figure 9c, the relationship between contact resistance and load is affected by loading and unloading conditions. It is shown that the number of cycles has the greatest impact on B, which is a very noteworthy phenomenon. Moreover, as the number of cycles increases, the values of the loading and unloading fitting parameter B gradually become closer.

### 4.2. Fitting and Discussion of the Effect of Enviroment Temperature on Contact Resistance

**Figure 10:**
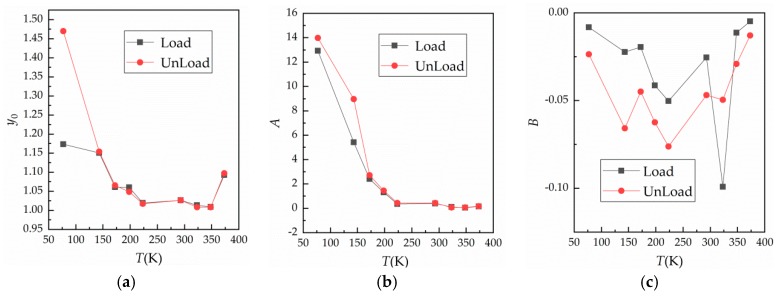
Relationship between fitting parameters and temperature. (**a**) Curve of $y_{0}$ against temperature; (**b**) curve of A against temperature; and (**c**) curve of B against temperature.

The curves shown in Figure 5a were fitted by Equation (3) and the variation of fitting parameters with temperature was obtained, as shown in Figure 10. 

In Figure 10a,b, at a temperature of 77 K (liquid nitrogen condition), $y_{0}$ changed a lot, reaching 1.1736 and 1.4699 at loading and unloading, respectively. This may be the result of liquid nitrogen insulation between the contact surfaces. When the temperature was lower than 293 K, the influence of temperature on $y_{0}$ and A was significant. In addition, $R_{0}$ changed considerably in the low temperature environment. At 293~345 K, fitting parameters $y_{0}$ and A changed very little, and were basically the same at loading and unloading, while $R_{0}$ also did not change much in this temperature range. At 373 K, the fitting parameter $y_{0}$ was higher than at 223~348 K. Based on this result, we suggest that surface oxidation accelerates, leading to rapid oxidation of the contact surface during the third loading cycle, thus making much higher than the first measured value. In Figure 10b, A decreases rapidly as the temperature rises from liquid nitrogen temperature to 223 K, but does not change much when the temperature continues to rise. Obviously, $y_{0}$ and A vary greatly at low temperature, so $R_{0}$ also varies significantly at low temperature. Further analysis showed that low temperature had a great influence on contact resistance $R_{0c\min}$ under maximum load, which is a factor that cannot be ignored in practical applications. In Figure 10c, parameter B at loading is higher than that at unloading, and with the change of temperature, it tends to decrease first and then increase. This indicates that contact resistance has a singularity with change of temperature, which is similar to experimental results reported in the literature [22]. However, due to surface roughness and the existence of an oxide film, the location of the singularity is transferred.

### 4.3. Fitting and Discussion of the Effect of the Number of the Contact Surfaces on Contact Resistance

**Figure 11:**
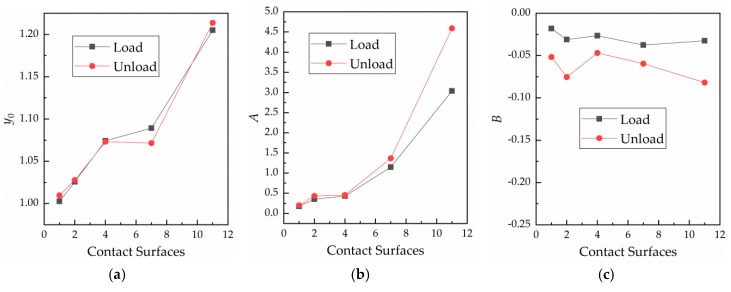
Relationships between the fitting parameters and number of contact surfaces. (**a**) Curve of $y_{0}$ against the number of contact surfaces; (**b**) curve of A against the number of contact surfaces; and (**c**) curve of B against the number of contact surfaces.

The curves shown in Figure 7 were fitted. In Figure 11, changes in the fitting parameters in response to variation of the number of contact surfaces are shown.

According to Figure 11, fitting parameter $y_{0}$ increases linearly with the number of contact surfaces, and the $y_{0}$ values remain basically the same at loading and unloading. Fitting parameter A increases with the number of contact surfaces. For instance, when the number of contact surfaces is 11, the value of A is notably large, as the contact becomes more unstable with the increase in the number of contact surfaces. In Figure 11c, parameter B at unloading is smaller than that at loading. Further, parameter B appears to remain relatively unchanged during loading as the number of contact surfaces varies. This shows that B is not affected by the number of contact surfaces, while the number of contact surfaces has a great influence on $R_{0}$.

## 5. Conclusions

The relationships between contact resistance and normal load, temperature and number of contact surfaces in terms of Cu bulks were experimentally studied. The relationship between contact resistance and normal load was fitted using the equation $R^{\;*}=y_{0}+Ae^{\;BF}$ under (i) a single contact surface at room temperature, (ii) temperatures ranging from 77 to 373 K, and (iii) a range of number of contact surfaces (1 to 11). The relationships between fitting parameters $y_{0}$, A and B with the number of cycles at room temperature under a single contact surface, the temperature changing from 77 to 373 K and a variable number of contact surfaces were also studied.

Experimental and fitting results showed that the contact electric resistance decreased rapidly with an increase in the normal force. Fitting parameter $y_{0}$ was found to be influenced by temperature and number of contact surfaces, but not by the cycle number. Specifically, in the low temperature zone, $y_{0}$ decreased with an increase in temperature. Further, as the number of interfaces was increased, $y_{0}$ increased almost linearly. The parameter A was not affected by the number of cycles, but it decreased quickly with an increase in temperature. Above 220 K, however, the parameter A remained unchanged. When the number of contact interfaces was increased, the value of A increased. Finally, parameter B increased slowly with the cycle number, but decreased only slightly and then increased with an increase in temperature. B was not affected by the number of contact interfaces.

In this work, the relationships revealed between contact electric resistance and normal load, number of cycles, environment temperature and number of contact interfaces can be used to predict variation trends of contact resistance in complex environments. Our results can also provide more accurate contact resistance parameters for calculating AC losses in superconducting cables and other electrical connectors. 

## Reference

## Figures and Tables

**Table 1:** Fitting of cyclic loading and unloading force against relative contact resistance of a single contact surface at room temperature.

Number of Cycles	Loading				Unloading			
	$y_{0}$	A	B	Range	$y_{0}$	A	B	Range
1	1.0132	0.5487	−0.0951	0.969	1.0040	0.1934	−0.0864	0.978
2	1.0014	0.1436	−0.0209	0.994	1.0080	0.2167	−0.0702	0.967
3	1.0028	0.1784	−0.0185	0.993	1.0098	0.1986	−0.0515	0.966
4	1.0052	0.1796	−0.0173	0.996	1.0123	0.1972	−0.0458	0.958
5	1.0070	0.1812	−0.0159	0.997	1.0143	0.1930	−0.0415	0.955
6	1.0087	0.1820	−0.0152	0.997	1.0160	0.1869	−0.0370	0.952
7	1.0110	0.1803	−0.0147	0.996	1.0187	0.1805	−0.0337	0.949
8	1.0127	0.1785	−0.0142	0.996	1.0206	0.1791	−0.0339	0.950
9	1.0135	0.1776	−0.0139	0.995	1.0208	0.1646	−0.0280	0.949
10	1.0144	0.1685	−0.0128	0.995	1.0224	0.1595	−0.0274	0.946
